# Immune mechanisms and medical comorbidity in ADHD: a narrative review

**DOI:** 10.1192/j.eurpsy.2025.173

**Published:** 2025-08-26

**Authors:** J. H. Newcorn

**Affiliations:** Psychiatry, Icahn School of Medicine at Mount Sinai, New York, United States

## Abstract

**Abstract: Introduction:**

The potential role of inflammatory and immune pathways in ADHD pathophysiology and pathogenesis has received considerable recent attention. Some of these pathways are long-studied in ADHD because of known or suspected mechanisms of high frequency comorbidities, such as obesity, sleep disorders, cardiovascular system disorders/migraine, atopic illnesses, and several autoimmune diseases. Aberrant cellular functioning, aberrant glial functioning, and/or aberrant mitochondrial dysfunction have all been implicated in mechanistic studies. While we separate these mechanisms conceptually, in reality they are intertwined and communicate with each other in a multi-directional complex of mechanisms.

**Objectives:**

To introduce the relationships among inflammation, immune mechanisms and ADHD, and provide a high level overview of current and cutting edge research in this area. We will review data linking ADHD with inflammation and immunologic mechanisms, recent findings on the genetic underpinnings of medical comorbidity in ADHD, and the relationship between maternal inflammation and genetic risk for ADHD in offspring.

**Methods:**

This is a focused, narrative review of recent literature in the field.

**Results:**

Epidemiological, genetic, and biomarker data converge to illuminate the pleiotropic role of immune mechanisms in the pathophysiology of ADHD. Suspected mechanisms include both innate and adaptive pathways. Epigenetic vulnerabilities interact with environmental factors to confer greater risk for health problems among individuals with ADHD. Likewise, many of these same risk factors also carry increased risk for ADHD.

**Conclusion:**

This information exemplifies the close relationship between psychiatric and medical conditions and/or their risk factors. The high heritability of the immune system and associated immunological dysfunctions provides a novel approach to understand the mechanisms through which gene-environment interactions may contribute to the occurrence and clinical presentation of ADHD.

**Disclosure of Interest:**

None Declared

